# Metabolomic Study of Aging in *fa*/*fa* Rats: Multiplatform Urine and Serum Analysis

**DOI:** 10.3390/metabo13040552

**Published:** 2023-04-12

**Authors:** Helena Pelantová, Petra Tomášová, Blanka Šedivá, Barbora Neprašová, Lucia Mráziková, Jaroslav Kuneš, Blanka Železná, Lenka Maletínská, Marek Kuzma

**Affiliations:** 1 Institute of Microbiology, Czech Academy of Sciences, 142 20 Prague, Czech Republic; 2 First Faculty of Medicine, Charles University and General University Hospital in Prague, 128 08 Prague, Czech Republic; 3 Faculty of Applied Sciences, University of West Bohemia, 306 14 Pilsen, Czech Republic; 4 Institute of Organic Chemistry and Biochemistry, Czech Academy of Sciences, 160 00 Prague, Czech Republic; 5 Institute of Physiology, Czech Academy of Sciences, 142 20 Prague, Czech Republic

**Keywords:** *fa*/*fa* rats, genetic obesity, metabolomics, NMR, LC-MS

## Abstract

Zucker fatty (*fa*/*fa*) rats represent a well-established and widely used model of genetic obesity. Because previous metabolomic studies have only been published for young *fa*/*fa* rats up to 20 weeks of age, which can be considered early maturity in male *fa*/*fa* rats, the aim of our work was to extend the metabolomic characterization to significantly older animals. Therefore, the urinary profiles of obese *fa*/*fa* rats and their lean controls were monitored using untargeted NMR metabolomics between 12 and 40 weeks of age. At the end of the experiment, the rats were also characterized by NMR and LC-MS serum analysis, which was supplemented by a targeted LC-MS analysis of serum bile acids and neurotransmitters. The urine analysis showed that most of the characteristic differences detected in young obese *fa*/*fa* rats persisted throughout the experiment, primarily through a decrease in microbial co-metabolite levels, the upregulation of the citrate cycle, and changes in nicotinamide metabolism compared with the age-related controls. The serum of 40-week-old obese rats showed a reduction in several bile acid conjugates and an increase in serotonin. Our study demonstrated that the *fa*/*fa* model of genetic obesity is stable up to 40 weeks of age and is therefore suitable for long-term experiments.

## 1. Introduction

Obesity represents a global epidemic problem with a complex etiology, including genetic and environmental factors [1]. The precise molecular mechanisms of obesity and its associated health problems are still under investigation. Good animal models are essential to better understanding the pathomechanisms of human disease [2].

Zucker fatty (*fa*/*fa*) rats are a well-established and widely used model of genetic obesity [3]. The mutation of a single recessive gene (*fa*) in the leptin receptor renders this gene nonfunctional and results in leptin sensitivity. *Fa*/*fa* rats develop obesity, hyperphagia, hyperinsulinemia, hyperlipidemia, and insulin resistance, although their glucose levels are normal. Since the seventies, this model of genetic-based early onset obesity has been frequently studied, along with the need to describe the background of obesity and its comorbidities at the molecular level.

Metabolomics is an extremely useful tool to monitor and explain metabolic disorders at the level of biochemical networks because the metabolome, in addition to the genetic predisposition, also reflects the actual physiological status of an organism as well as the influence of various external factors [4,5]. Currently, two leading analytical platforms are preferred in metabolomics: nuclear magnetic resonance (NMR) spectroscopy and mass spectrometry (MS), in which MS is usually combined with the separation step. Both techniques provide complementary results and thus together enable a more comprehensive view of the metabolic profile of samples under study.

Several publications using a Zucker fatty rat metabolomic characterization have been published in the last two decades, and they have involved different biological matrices and analytical platforms. Urinary profiles were studied by NMR [6,7], NMR and HPLC-MS [8,9], or UPLC-MS [10]. Plasma samples were characterized using NMR [11], GC-MS [12], UPLC-MS [13], or a combination of NMR, UPLC-MS, and GC-MS [14]. Waldram et al. characterized the microbiome in parallel with urine and plasma samples [15]. The metabolic composition of urine, blood, and fecal water was analyzed by Phetcharaburanin et al. [16]. Lees et al. performed a complex NMR-based multiple matrix analysis of urine, plasma, liver, kidney, and pancreas samples [17]. Recently, Melnyk et al. compared serum profiles of obese and lean Zucker fatty rats using a combination of HPLC-UV, HPLC-ECD, and LC-MS analytical platforms [18]. A common feature of all the above-mentioned papers was the young age of the experimental animals, which were followed up to a maximum of 20 weeks of age. To the best of our knowledge, no metabolomic study of *fa*/*fa* rats over a wider age range has been published to date. The main aim of our study was to cover the gap in the metabolomics data of older *fa*/*fa* rats in long-term studies. It is necessary to verify whether the specific features in *fa*/*fa* urinary and plasma metabolic profiles revealed in young rats persist during aging.

A few years ago, a study focused on 12–32 weeks old Zucker fatty rats showed that aging and obesity significantly contributed to increased peripheral insulin resistance, which further worsened the activation of the hippocampal insulin signaling cascade [19]. Therefore, the authors hypothesized that aged *fa*/*fa* rats might be a suitable model to study the relationship between metabolic and neurodegenerative disorders. A recently published study [20] aimed to investigate the potential neuroprotective effects of a newly developed palmitoylated analog of prolactin-releasing peptide (palm^11^-PrRP) [20,21] in a model of aged *fa*/*fa* rats.

For our study, a recently published model [20] was utilized and both urine and serum samples from examined animals were collected for complete metabolomic characterization. Our approach was based on the untargeted NMR-based long-term monitoring of urine and was supplemented by NMR and LC-MS serum analysis of rats at 40 weeks of age. In addition, this study was the first targeted analysis of serum bile acids and neurotransmitters in Zucker fatty rats. To the best of our knowledge, the present study provides the first characterization of Zucker fatty rats aged 12 to 40 weeks using NMR- and MS-based metabolomics of urine and serum.

## 2. Experimental Design

### 2.1. Experimental Animals

All the animal experiments were performed by following the ethical guidelines for work with animals by the Act of the Czech Republic No. 246/1992 and were approved by the Committee for Experiments with Laboratory Animals of the CAS. The experiments were conducted on homozygous Zucker *fa*/*fa* male rats (*fa*/*fa*) and their lean counterparts *fa*/^+−^ (control) rats. Six-week-old male rats of both genotypes were purchased from Charles River (Lecco, Italy). The rats were provided with a standard Ssniff diet (Spezialdiäten GmbH, Soest, Germany) (58% carbohydrates, 9% fat, and 33% protein) and water ad libitum. The animals were on a 12:12-h light:dark cycle (lights on from 5:00) and maintained at a constant temperature of 22 ± 2 °C.

*Fa*/*fa* rats and their lean controls were aged to 32 weeks of age. From this time point on, Mráziková et al. studied the impact of palm^11^-PrRP infused for 2 months on a newly established group of *fa*/*fa* rats using Alzet osmotic pumps. The existing *fa*/*fa* and lean control groups, whose urinary metabolic profiles were continuously monitored from 12 weeks of age on, were infused with saline.

### 2.2. Study Design and Sample Collection

**Figure 1:**
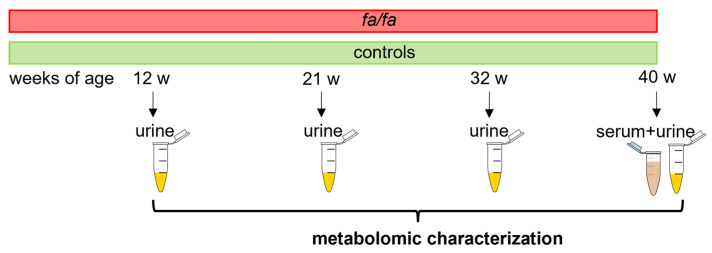
Experimental design.

The metabolomic characterization of the aged *fa*/*fa* rats was based on an NMR analysis of urinary data collected from *fa*/*fa* and control rats at 12, 21, 32, and 40 weeks of age. The model description was completed using untargeted NMR and LC-MS analyses of serum samples from saline-treated *fa*/*fa* and control groups acquired at the end of the treatment period (Figure 1).

Rats at 12, 21, 32, and 40 weeks of age were placed in individual metabolic cages (Tecniplast, Buguggiate, Italy) with free access to water but not food. Urine samples were collected overnight (from 5 pm to 8 am) with added NaN_3_ and then stored at −80 °C.

At the end of the experiment, overnight fasted rats were anesthetized by pentobarbital (60 mg/kg), and their blood was obtained from the abdominal aorta. Plasma and serum samples were stored at −80 °C until processing.

### 2.3. Biochemical Parameters

The concentration of fasting plasma insulin was measured by RIA assay, and the leptin concentrations were measured by ELISA (Millipore, St. Charles, MI, USA). Colorimetric assays were used to determine the plasma levels of cholesterol (CHOL) and triglycerides (TG) (Erba Lachema, Brno, Czech Republic). All the measurements were performed according to the manufacturer’s instructions.

### 2.4. NMR Sample Preparation and Experiments

Prior to NMR analysis, urine samples were thawed at room temperature and centrifuged at 18.620× *g* for 5 min. A 200 µL volume of supernatant was mixed with 340 µL H_2_O and 60 µL phosphate buffer (1.5 M KH_2_PO_4_ in D_2_O containing 2 mM NaN_3_ and 0.1% trimethylsilyl propionic acid (TSP), pH 7.4) to reach a H_2_O:D_2_O ratio of 9:1 and was then transferred to a 5-mm NMR tube.

A 220 µL aliquot of serum sample was mixed with 440 µL cold methanol. The mixture was kept in a freezer at −20 °C for 30 min and then centrifuged at 18.620× *g* for 10 min at 4 °C. The supernatant was transferred into a fresh vial and vacuum-dried. The evaporated supernatant was dissolved in 450 µL D_2_O with 50 µL 1.5 M phosphate buffer and then transferred into a 5 mm NMR tube.

The NMR data were acquired on a 600 MHz Bruker Avance III spectrometer (Bruker BioSpin, Rheinstetten, Germany) equipped with a 5 mm TCI cryogenic probe head. All the experiments were performed using Topspin 3.5 software at 300 K with automatic tuning and matching, shimming, and the adjustment of the 90° pulse length for each sample. The proton spectra of both urine and serum samples were acquired using a Carr-Purcell-Meiboom-Gill (CPMG) pulse program (cpmgpr1d) with presaturation during relaxation delay d1 (4 s) with the following parameters: number of scans (NS) = 48 for urine, NS = 256 for serum; number of data points (TD) = 64 k; spectral width (SW) = 20 ppm; echo time = 0.3 ms; and loop for T2 filter = 126. The CPMG experiment was chosen to suppress broad resonances of major urinary proteins that occur naturally in rodent urine [22] and to improve the baseline close to lipid signals in the serum extracts. A short *J*-resolved experiment (for both urine and serum NS = 2, SW = 16 ppm, TD = 8 k, number of increments = 40, and d1 = 2 s) was performed on each sample to facilitate metabolite identification. The metabolite assignment was supported by the information extracted from the HSQC and TOCSY spectra acquired for the selected samples.

The raw spectral data were processed using TopSpin 3.5 software (Bruker BioSpin, Rheinstetten, Germany). Free induction decays (FIDs) were multiplied by an exponential window function (LB = 0.3 Hz) before the Fourier transformation and were automatically phased. Because the TSP signal was slightly shifted and broadened by nonspecific binding with proteins, the spectra were referenced to the downfield peak of the alanine doublet at 1.492 ppm in urine and the downfield peak of the α-glucose doublet at 5.245 ppm in serum extracts. Regions with water, urea (in urine), and methanol (in serum extracts) signals were excluded. Spectra within the 0.2 to 10.0 ppm range were normalized using the probabilistic quotient normalization (PQN) method [23] with the pooled control group as a standard.

### 2.5. MS Sample Preparation and Experiments

For the untargeted serum analysis, a 50 μL serum sample was mixed with a 5 μL internal 4-chlorophenyl-alanine standard (1 mg/mL) and a 200 μL acetonitrile/methanol mixture (3/5, *v*/*v*). The sample was kept in a freezer at −20 °C for 30 min and then centrifuged at 7700× *g* for 10 min at 4 °C. The injection volume of the supernatant was 10 µL. Quality control samples were prepared by mixing 5 μL serum aliquots of all the samples, which were then processed in the same manner. To evaluate the entire process, blank samples were prepared and then underwent the same process as real samples. The extracts were separated by HPLC (Agilent 1200 LC, Agilent Technologies, Santa Clara, CA, USA) equipped with an Intrada amino acid column (150 mm × 2 mm, 3 μm, Imtakt, Portland, OR, USA). Mass detection was performed with a mass spectrometer (micrOTOF-Q III, Bruker Daltonics, Billerica, MA, USA). The separation and detection conditions in positive and negative modes were set up according to a previously published method [24]. Blank samples and quality control samples were analyzed together with the clinical samples. Samples were measured in random order, and they were interrupted by blanks and quality control samples. The data were recalibrated for exact mass, converted in DataAnalysis 4.2 (Bruker), and then imported into MZmine 2.23 software. Our previously published paper described the data analysis procedure in detail [25]. The data were normalized by the total intensity.

A targeted analysis of bile acids and neurotransmitters was performed according to a previously optimized method [26,27]. The preparation of neurotransmitter derivates suitable for MS analysis was performed according to the instructions in the EZ: fast kit user manual (Phenomenex, Torrance, CA, USA). The bile acids were extracted from 50 µL serum using 160 µL acetonitrile. The supernatant was evaporated and reconstructed in 50 µL methanol-water mixture (1:1, *v*/*v*). Bile acids were separated on an HPLC system (Dionex Ultimate 3000, Dionex Softron GmbH, Germering, Germany) equipped with a Hypersil GOLD column (150 × 2.1 mm, 3 µm, Thermo Fisher Scientific, Inc., Waltham, MA, USA) and a SecurityGuard column (Phenomenex, Torrance, CA, USA) and detected in a triple quadrupole mass spectrometer (TSQ Quantum Access Max with H-ESI II probe, Thermo Fisher Scientific, Inc., Waltham, MA, USA) operating in SRM mode. The parameters of the separation and detection are described in [26]. The peak area of individual metabolites was normalized by finding the area of the corresponding internal standards.

### 2.6. Statistical Analysis

Untargeted multivariate analysis, which was based on the analysis of equidistantly binned spectra (bin width = 0.01 ppm) in NMR and on the analysis of all signals above the intensity threshold for MS, was performed in Metaboanalyst 4.0 software [28]. A principal component analysis (PCA) on Pareto-scaled data was used to monitor sample grouping and detect potential outliers. The statistical model was built using partial least squares-discriminant analysis (PLS-DA) and then validated by leave-one-out cross-validation (LOOCV) and permutation tests. The results of the PLS-DA models were evaluated using variable importance in projection (VIP) scores, which identified the important bins contributing the most to the groups’ separation.

A univariate analysis was performed using MATLAB software (version 9.10 R2021a). The application of the standard Student’s two-sample *t*-test was based on the result of the Lilliefors test for normality; the *p*-value cutoff was 0.05. In NMR metabolomics, all well-resolved non-overlapping signals or parts of multiplets were subjected to analysis. The identification of individual metabolites in the NMR spectra was made using Chenomx NMR Suite software (Chenomx Inc., Edmonton, AB, Canada) or previously published data and confirmed by the comparison of proton and carbon data acquired for selected samples with the Human Metabolome Database (HMDB [29]) and the Biological Magnetic Resonance Bank (BMRB [30]) databases (Table S1 and Figures S1 and S2 in Supplementary Data). The MS data analysis procedure was described in detail in our previously published paper [25]. The identification of significantly changed metabolites in the MS spectra was based on the exact mass, isotope pattern, MS/MS fragments, and retention time. The detected parameters were compared with standards, databases (HMDB, MetFrag [31]), and previously published data (Table S2 in Supplementary Data).

## 3. Results

### 3.1. Morphometric and Biochemical Parameters

In our recent study, we published the results of several rat morphometric and metabolic parameters at 32 and 40 weeks of age. Here, we present data on the parameters that significantly changed at the end of the experiment covering the time range of 12–40 weeks (Table 1). The body weight was significantly higher in the *fa*/*fa* rats than in the controls during the entire monitored period. Similarly, the plasma concentrations of insulin, leptin, cholesterol, and triglycerides were significantly raised in the *fa*/*fa* rats compared with the control rats at each sample collection time.

### 3.2. NMR-Based fa/fa Model Characterization Using Urinary Metabolic Profiles

**Figure 2:**
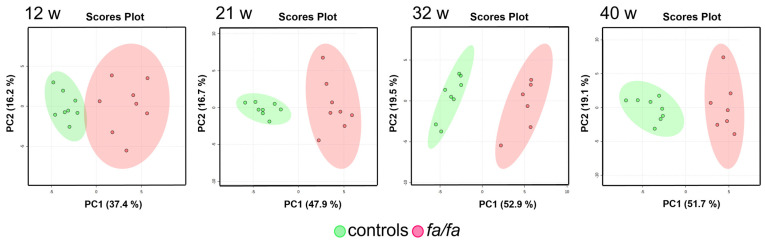
Score plots of PCA models of urine at 12, 21, 32, and 40 weeks. *Fa*/*fa* group is marked in red, control group in green.

The main advantage of urine-based metabolomics is the easy and noninvasive sample collection, which enables the continuous monitoring of metabolic alterations over time. During the first step, the urinary metabolic profiles of the *fa*/*fa* and control rats were compared at each sample collection time, i.e., at 12, 21, 32, and 40 weeks of age. An untargeted multivariate analysis was performed on the binned spectra to explore the distribution of the samples, to build appropriate models, and to identify the spectral regions that contributed the most to the group separation. Unsupervised PCA did not detect any outliers and showed clear differences between the *fa*/*fa* and control groups at all time points (Figure 2).

PLS-DA models were then built and evaluated (Figure S3 in Supplementary Data). LOOCV was used to assess the quality of various models via the computed parameters R2 and Q2. The interpretation of the PLS-DA models was based on the examination of VIP scores; the most important bins (those with VIP scores greater than 2.0) were considered. The assignment of these bins revealed similar sets of metabolic changes in all four models, contributing the most to the separation of the *fa*/*fa* and control groups: they showed elevated levels of citrate, 2-oxoglutarate, fumarate, malate, and allantoin and decreased concentrations of creatinine, taurine, and hippurate. Nevertheless, the high *p*-values of the permutation tests indicated possible model overfitting due to the small number of samples, so the PLS-DA outputs can only be considered approximate.

Univariate analysis was subsequently used to evaluate variations in individual metabolite levels. A parametric Student’s *t*-test was performed on the set of seventy signals in the urine spectra and revealed primarily increased levels of tricarboxylic acid (TCA) cycle metabolites, lactate, choline, glycine, alanine, 1-methylnicotinamide, and trigonelline, and attenuated levels of microbial co-metabolites, creatinine, taurine, formate, methylsuccinate, 1-methyl-4-pyridone-3-carboxamide (4-PY), and lipid species (Table 2).

**Figure 3:**
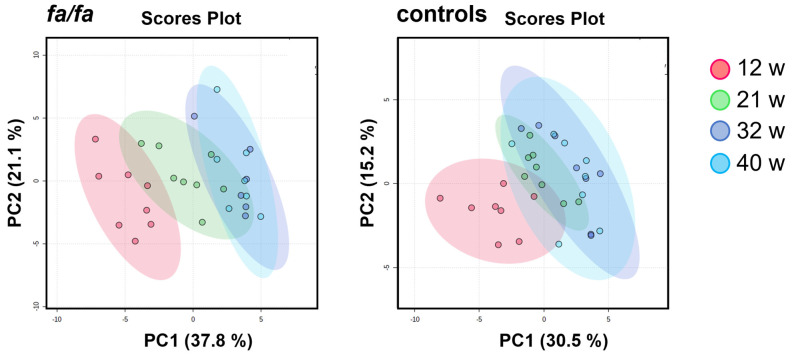
Score plots of PCA models of urinary profile development during aging, generated separately for *fa*/*fa* and control rats. Samples collected at 12 weeks are marked in red, at 21 weeks in green, at 32 weeks in blue, and at 40 weeks in turquoise.

Another approach that can expand the comprehensive view of aging in Zucker fatty rats is to track variations in individual metabolites over time. PCA models built for the *fa*/*fa* and control groups separately from all time points clearly show a gradual change in urinary profiles during the experiment (Figure 3). This shift, which is most visible between 12 and 21 weeks of age, seems to be more intensive in *fa*/*fa* rats than in control rats.

Table 3 summarizes the significant metabolic changes observed between successive sampling points from 12 to 40 weeks of age, as evaluated by paired *t*-test in parallel for *fa*/*fa* rats and their lean controls.

**Figure 4:**
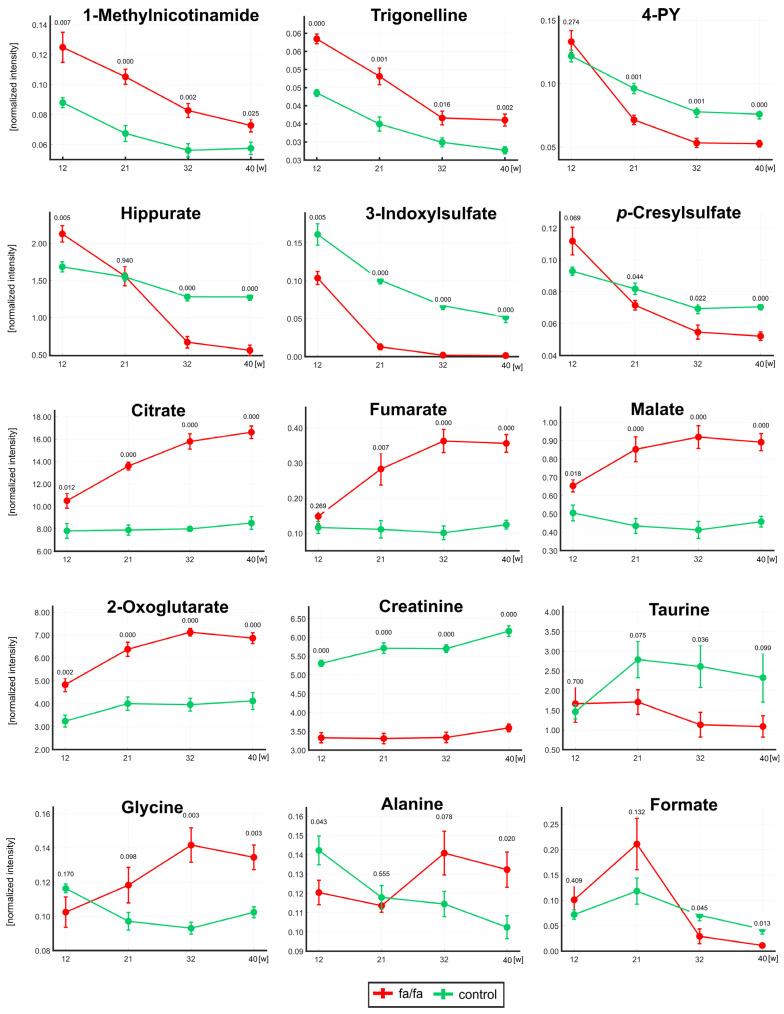
The development of normalized intensities for selected metabolites at 12, 21, 32, and 40 weeks. The statistical significance was analyzed by Student’s *t*-test (*fa*/*fa* vs. controls). *p*-values and standard deviation error bars are given for each collection time. *Fa*/*fa* rats are marked in red, controls in green. 4PY—1-Methyl-4-pyridone-3-carboxamide.

The table reveals some similar trends in the *fa*/*fa* and control strains: a significant decrease in nicotinamide metabolites and microbial co-metabolites and altered levels of allantoin and pseudouridine. However, a significant increase in TCA metabolites was observed only in *fa*/*fa* rats up to 32 weeks of age, and an increase in taurine and a decrease in choline and glycine levels were observed only in young lean controls. The evolution in the concentrations of selected important metabolites is well illustrated by the curves in Figure 4.

### 3.3. NMR- and MS-Based fa/fa Model Characterization Using Serum Metabolic Profiles

The serum samples for the *fa*/*fa* model characterization were collected at the end of the experiment from saline-treated *fa*/*fa* and control rats. The acquired NMR and MS data were evaluated using a procedure analogous to that used for the urine samples.

**Figure 5:**
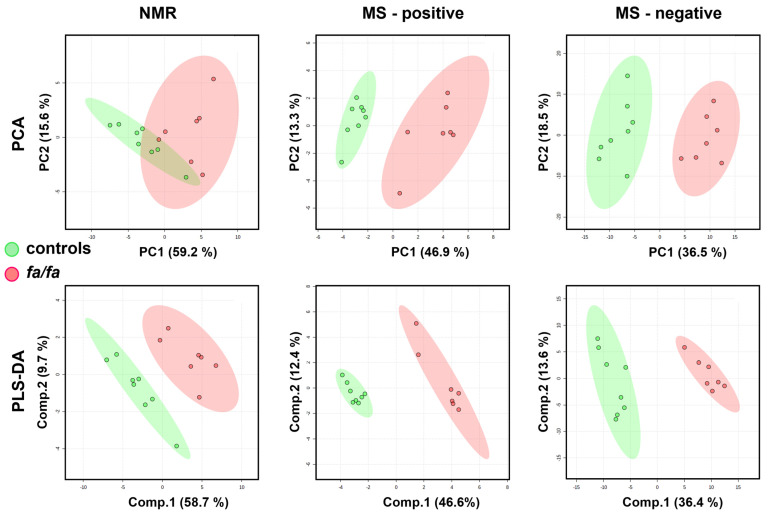
Score plots of PCA and PLS-DA models of serum from *fa*/*fa* and control rats (at 40 weeks of age) based on NMR and MS data. The LOOV results for 3 principal components: accuracy = 1, R2 = 0.98, Q2 = 0.89 for NMR-based; accuracy = 1, R2 = 0.99, Q2 = 0.96 for positive-MS-based; accuracy = 1, R2 = 0.99, Q2 = 0.92 for negative-MS-based. *Fa*/*fa* group is marked in red, control group in green.

The whole spectra were first subjected to multivariate analysis (Figure 5). The trend in group separation observed in the PCA appeared more pronounced in the supervised PLS-DA model, which was satisfactorily validated using the LOOCV method. Bins with VIP values > 2 correspond to increased lactate, alanine, citrate, and lipids and decreased levels of glucose, creatine, 3-OH butyrate, glutamine, valine, and leucine in the NMR-based model. In the MS analysis, high VIP values indicated reduced levels of carnitine and valine and altered levels of several phosphocholines (PC 36:2, 36:4, and 38:4) and lysophosphatidylcholines (LysoPC 16:1, 18:2, and 20:4) as the most discriminating combination of serum metabolites. Unfortunately, as in the case of the urine, the poor permutation test results for both NMR and MS models suggest a possible risk of model overfitting.

Univariate statistical analysis using the parametric Student’s *t*-test was applied to fifty-four NMR signals representing thirty-nine metabolites. Increased levels of TCA cycle metabolites, lactate, glycerol, alanine, allantoin, and lipids and decreased concentrations of glucose, arabinose, creatine, choline, amino acids, and hydroxy acids were detected in the *fa*/*fa* group compared with the controls. The MS data were measured in negative and positive ionization modes, but significantly altered metabolites were identified and quantified only in the positive mode. The univariate analysis of the MS data revealed lowered serum amino acids, carnitine, creatine, and deoxycytidine, increased PC levels (except decreased PC (36:2)), and altered concentrations of LysoPCs in the *fa*/*fa* rats (Table 4).

The targeted LC-MS analysis of seventeen bile acids in serum showed a significant decrease in seven of them. Of the five neurotransmitters analyzed here, only the serotonin levels increased significantly in obese *fa*/*fa* animals compared with the lean controls (Table 5).

## 4. Discussion

The main objective of this study was the metabolomic characterization of Zucker fatty rats during aging over a long-time scale of 10 months since all previous metabolomic research on *fa*/*fa* rats has only included animals up to 20 weeks of age. Therefore, our task was to verify whether the differences detected in young *fa*/*fa* rats persist up to 10 months of age and thus prove the suitability of this genetic model of obesity for long-term studies. Our research was primarily based on urine analysis because its non-invasive collection allows for the continuous monitoring of the animals without interfering with their metabolism. The untargeted analysis of serum samples obtained at the end of the experiment, together with the targeted analysis of serum bile acids and neurotransmitters, completed the metabolomic characterization of the studied model. The phenotype of our *fa*/*fa* model was verified from acquired morphometric and biochemical data. They demonstrated that *fa*/*fa* rats developed obesity, mild glucose intolerance, and mild central and peripheral insulin resistance in our study [20].

The first urine analysis of 12-week-old animals corresponds to the approximate age of *fa*/*fa* rats used in other studies [6,8,11,17]. The next model at 21 weeks of age correlates with the maximum age of experimental animals in previously published metabolomic papers [9,10,13,14]. The subsequent sample collections and analyses at 32 and 40 weeks of age then extended the monitoring of the animals for twice as long. All the detected changes in metabolic profiles described in our study were compared with those reported in the published data for younger Zucker fatty rats or other rodent models of obesity.

The first finding in our study was the significant decrease in the host-microbial aromatic metabolites hippurate, phenylacetylglycine, 3-indoxylsulfate, and *p*-cresylglucuronide in the *fa*/*fa* group compared with its lean control, indicating the altered microbial metabolism of aromatic compounds in *fa*/*fa* rats. Analogous urinary changes were presented in previous studies of Zucker obese rats [6,15,16,17], in rodent models with diet-induced obesity such as spontaneously hypertensive or Wistar Kyoto (WKY) rats [32] and C57BL/6J mice [33], and in obese insulin-resistant humans [34].

In a human study, Brial et al. showed that urine hippurate is positively associated with microbial gene richness and can be used as a marker of metabolic health [35]. In the next paper, based on data from the Study of Health in Pomerania [36], the authors detected significant genus-metabolite associations for hippurate, succinate, indoxyl sulfate, and formate and the association between gut microbiome alpha diversity and levels of hippurate, formate, and 4-hydroxyphenylacetate.

The monitoring of urinary profiles over 40 weeks showed a rapid decline in microbial co-metabolite concentrations with age (Figure 4), which explains some of the different results observed in much younger *fa*/*fa* rats. The hippurate levels at 12 weeks of age were significantly higher in *fa*/*fa* rats than in control rats, which is consistent with the finding in 12-week-old Zucker fatty rats by Williams et al. [8]; the same group of authors described a negative correlation of hippurate with age between 4 and 20 weeks [9]. After a sharp decrease between the first two samplings, 3-indoxyl sulfate was not detected at all in *fa*/*fa* rats from 32 weeks of age. Formate, another product of gut microbial origin, was significantly associated with body mass index in the human INTERMAP study [37]. Higher urinary formate in *fa*/*fa* rats compared with lean controls was reported until 12 weeks of age [6] and at 14 weeks [17]. In our study, formate was insignificantly higher at 12 and 21 weeks in the *fa*/*fa* group; a significant decrease compared with lean rats was detected from 32 weeks of age. It should be considered that the age of 12 weeks corresponds to puberty and 21 weeks to early maturity in male Zucker fatty rats [38]. The dynamic evolution of the gut microbiota composition during sexual maturation has been reported repeatedly in humans [39,40,41]. Recently, the shift in gut bacteria during sexual maturity in Sprague-Dawley rats was explored [42]. Lees et al. published that age is a major contributor to the microbiome composition in *fa*/*fa* rats aged 5–14 weeks [43]. In summary, the significant changes in microbial co-metabolite concentrations observed in our study in rats aged 12 and 21 weeks old may be directly associated with the development of their gut microbiome during sexual maturation.

Nicotinamide metabolism is the next pathway that was significantly altered in the obese *fa*/*fa* group. Although these changes in *fa*/*fa* rats have only been discussed in a single study [6], the significant increase in 1-methylnicotinamide and its metabolites in obese animals is a typical feature described in many rodent models: in a diet-induced obesity mouse model [44,45], in a diet-induced obesity model of WKY and spontaneously hypertensive rats [32], in a monosodium glutamate (MSG)-induced obesity mouse model [46] and in genetically obese *db*/*db* mice [6]. The last above-mentioned paper identified 1-methylnicotinamide as a unique biomarker for monitoring diabetes and obesity. The significant changes in nicotinamide metabolites can be easily underestimated for two reasons. First, their signal intensities may be biased by the broad background of major urinary proteins if proton spectra are acquired using the 1D-NOESY pulse sequence [22]. Second, changes in these minor metabolites do not contribute enough to the complex multivariate statistical model to be reflected in the VIP values [45] and are detected primarily by the univariate targeted approach.

Our study is the first to describe a significant reduction in putrescine, a metabolite of polyamine metabolism, in *fa*/*fa* rats. The reduced excretion of putrescine, which might be related to the accumulation of white adipose tissue and obesity development, was also reported in mice with MSG-induced obesity [46] and in WKY with diet-induced obesity [47].

In the current study, we observed elevated levels of lactate and alanine in the serum and urine and pyruvate in the serum. These changes could be triggered by a disorder in the mitochondrial respiratory chain system. Under anaerobic conditions, pyruvate is converted to lactate through lactate dehydrogenase. An abnormal accumulation of lactate was reported in the blood and urine of *fa*/*fa* rats [6,11,15] and in the blood and urine of DIO mice [48]. An unusual accumulation of pyruvate was also reported in rats with diet-induced obesity [49] probably due to the inhibition of pyruvate dehydrogenase. Alanine can be produced from pyruvate via alanine transaminase. The urinary alanine concentration ratios between the *fa*/*fa* and control groups varied significantly during our study. At 12 weeks, this level was significantly higher in the controls, while from 32 weeks of age on, urinary alanine was predominant in the *fa*/*fa* group. Consistent with this trend, significant reductions in alanine were observed in the peripheral venous blood of *fa*/*fa* rats at 4 weeks of age [16] and in *fa*/*fa* rat urine at 8 weeks of age [7].

The elevated levels of citrate and fumarate in urine and serum, together with increased urinary malate and 2-oxoglutarate levels, suggest the upregulation of the TCA cycle in obese animals, which may be caused by an excess of the TCA substrate pyruvate. Our findings are consistent with those found in Zucker obese rats at 10, 12, or 14 weeks of age [6,15,17]. Salek et al. linked the increase in TCA intermediates to hyperglycemia-induced systemic stress. Lees et al. explained this finding by noting the different energy expenditure and utilization in obese and lean Zucker rats. Urinary monitoring during the experiment showed that while TCA metabolite levels in *fa*/*fa* rats gradually increased until 32 weeks of age, whereas they remained almost stable in the lean controls.

Except for alanine, the serum levels of several amino acids decreased in *fa*/*fa* rats. Surprisingly, significantly lower concentrations of the branched-chain amino acids (BCAAs) valine and leucine were found in the serum of the *fa*/*fa* group compared with the lean controls, which was confirmed independently by both NMR and LC-MS analysis. An analogous decrease in plasma valine and leucine was also observed in our recent WKY rat model when the animals were fed a high fat (HF) diet [47]. Park et al. detected a decrease in circulating BCAAs in C57BL/6N mice fed an HF diet [50]. These findings contrast with most previous publications reporting elevated BCAA levels as a significant marker of obesity and diabetes [51]. She et al. reported elevated plasma BCAAs in 11-week-old obese Zucker rats [52]. Reduced levels of serum valine and leucine in our study have been independently confirmed by both NMR and LC-MS analysis, but unfortunately, we do not yet have a satisfactory explanation for them. A partial explanation for the different results may be related to increased levels of alanine, which is a product of BCAA catabolism in muscle. Dunn and Hartsook observed that obese Zucker fatty rats had a higher rate of protein muscle breakdown and were less efficient at retaining amino acids that had been incorporated into muscle [53].

Obese Zucker rats have significantly attenuated serum creatine and urinary creatinine levels, consistent with previous studies in this model [15,16]. Reduced creatinine levels in *fa*/*fa* obese rats have also been reported by Salek and Lees [6,17]. Creatinine is formed from creatine phosphate in muscle. Serum and urine creatinine were positively correlated with muscle mass and body weight, with a greater degree of correlation with muscle mass [54,55]. Lower urinary creatinine levels can be explained by high body weight, lack of muscle mass, and low physical activity in obese *fa*/*fa* animals.

In the untargeted LC-MS analysis of serum, in addition to the decrease in amino acid concentrations also observed in the NMR analysis, we detected altered serum levels of several phosphatidylcholines (PCs) and lysophosphatidylcholines (LysoPCs). LysoPC (17:0) and LysoPC (18:2) were significantly decreased in *fa*/*fa* serum compared with the lean controls. Bao et al. found that LysoPC (17:0) reduced blood glucose and alleviated insulin resistance and related metabolic disorders in HF diet-induced mice by activating glucagon-like peptide 1 and promoting insulin secretion [56]. Plasma LysoPC (18:2) was negatively correlated with the insulin resistance index in a study of healthy, prediabetic, and type 2 diabetic individuals [57]. In the population-based Cooperative Health Research in the Region of Augsburg (KORA) cohort, adults with low serum LysoPC (18:2) had a greater risk of developing impaired glucose tolerance over seven years of follow-up [58]. Low plasma LysoPC (18:2) was also identified as an independent predictor of the incidence of type 2 diabetes in a cohort of Finnish men [59].

Notably, we observed increased levels of LysoPC (14:0) and LysoPC (20:4), which is the precursor for the inflammatory mediators eicosanoids [60]. Overweight/obese subjects showed higher levels of LysoPC (C14:0) and LysoPC (C18:0) and lower levels of LysoPC (C18:1) than lean subjects [61]. Significantly increased concentrations of PC (38:4) and PC (40:6) in our model were also detected in fat-fed mice and were positively correlated with fasting glucose [62]. The concentration of PC (32:1) in *fa*/*fa* rats in our study was twice that of the controls. The opposite result was observed by Gowda et al., namely, a significant decrease in PC (32:1) in rats fed an HF diet was negatively associated with obesity [63].

The LC-MS analysis of the serum also revealed decreased levels of carnitine and increased acylcarnitine (C18:1) in the *fa*/*fa* group compared with the control group. Carnitine is a key metabolite for transporting long-chain fatty acids in the form of acylcarnitines to mitochondria. Its low concentration is often associated with obesity [61], and its supplementation can have a positive effect on weight loss [64]. Increased concentrations of acylcarnitines (C18:1), (C14:1), and (C14:2) were also detected in overweight patients with impaired glucose tolerance [65].

The concentrations of seventeen bile acids (BAs) in the serum of the *fa*/*fa* and control rats were analyzed using a targeted LC-MS approach. We found significantly reduced levels of all five glycine conjugates studied here, together with two taurine conjugates of BAs. The BA concentrations in the blood, liver, or fecal extracts have often been studied in human and experimental models in relation to obesity and diabetes, but with controversial results. The decrease in several conjugated BAs observed in our study can be attributed to the perturbated metabolism of BAs and indicate dysregulation of lipid and carbohydrate metabolism, energy expenditure, and the presence of inflammation [66]. In addition, it should be considered that the level of circulating BAs could also be affected by the higher age of the animals under study [67], as was described previously in mice [68] and humans [69].

The conjugation of primary BAs takes place in the liver, from which they are released via the gallbladder into the intestine. The gut microbiome is responsible for their deconjugation and conversion to secondary BA. Both primary and secondary BAs are subsequently re-conjugated with glycine or taurine in the liver and released back into the gut. Thus, the concentration of conjugated BAs has also been modified by the gut microbiome [70]. For instance, the comparison of germ-free and conventional mice showed different sizes and compositions of the BA pool [71]. An analysis of the fecal microbial composition in 10-week-old obese and lean Zucker rats revealed different relative abundances of the dominant members of their intestinal microbiota [15]. Later, Lees et al. highlighted that the gut microbiota composition may be more influenced by age and the cage environment than the genotype [43]. Hakkak et al. analyzed the fecal microbiome of genetically obese and lean Zucker rats housed individually in cages [72]. Clear differences in intestinal microbiota populations associated with both the time point of the study and the lean or obese status were reported. Thus, based on our observed changes in urinary microbial co-metabolite levels, we can speculate that the different concentrations of conjugated BA between the *fa*/*fa* and control groups may be attributable to differences in the composition and activity of the gut microbiome.

A targeted LC-MS analysis of five selected neurotransmitters (γ-aminobutyric acid, kynurenine, serotonin, hydroxytryptophan, and tyramine) in serum showed significantly higher serotonin concentration in obese *fa*/*fa* rats. All serotonin-related studies in Zucker rats to date have focused on the serotogenic mechanism in the brain. Because serotonin is unable to cross the blood-brain barrier, there are two independent pools with opposing functions in the regulation of energy homeostasis. An increase in central serotonin, which is made in the neurons of the brainstem, is expected to decrease body weight, whereas the increasing activity of peripheral serotonin, which is produced primarily in the gut, increases body weight and adiposity [73,74].

Significantly higher serum serotonin was reported in HF diet-fed mice than in lean controls [75]. Since diet-induced obesity is associated with inflammation, the authors hypothesized that blood serotonin could serve as an important mediator of inflammation. The majority of serotonin in the body is synthesized in the periphery within the gut neurons and enterochromaffin cells (ECs) by the enzyme tryptophan hydroxylase Tph1. Thp1 activity is positively regulated by microbiota-derived short-chain fatty acids (SCFAs) or glucose [76]. Serotonin from the gastrointestinal tract is absorbed by enterocytes or can enter the bloodstream, where platelets absorb it by serotonin reuptake transporter (SERT) [77]. Bertrand et al. reported increased serotonin in the ileum in western-type diet-fed rats caused by increased ECs and mRNA for Tph1 enzyme [78]. It was accompanied by a decrease in SERT mRNA and protein, which prevents serotonin reuptake. Crane et al. found that *Tph1*-deficient mice fed the HF diet were protected from obesity, insulin resistance, and nonalcoholic fatty liver disease while exhibiting greater energy expenditure through their brown adipose tissue. The authors speculated that the inhibition of serotonin signaling or its synthesis in adipose tissue might be an effective treatment for obesity and its comorbidities [79]. Another study of mice on the HF diet indicated that the intraperitoneal injection of serotonin prevented obesity by inducing an increase in the activity of mitochondria and an elevation of energy metabolism in skeletal muscle [80]. We can speculate that the increased serotonin level in the plasma of *fa*/*fa* rats could be caused by its increased synthesis in the gastrointestinal tract and limited absorption in the gut and by platelets.

It should be added that our study has similar limitations to other experimental models of obesity. The results obtained are to some extent influenced by the way obesity was induced (type of diet, genetic background). In addition, we have documented in this paper that even using an identical *fa*/*fa* rat model, the levels of many metabolites are significantly affected by the age of the animals. Furthermore, not all the findings described in the experimental model can be easily transferred to human obesity. On the other hand, we verified certain typical trends observed across all experimental models. An example is the altered metabolism of niacinamide, where an increase in 1-methylnicotinamide was also observed in the urine and serum of obese humans [6,81]. Nicotinamide *N*-methyltransferase, which catalyzes methylation of niacinamide to generate 1-methylnicotinamide, has been proposed as a promising therapeutic target to prevent or treat obesity and diabetes [82,83].

## 5. Conclusions

In this study, the metabolic profiles of obese and lean *fa*/*fa* rats were monitored for the first time on a long-term basis. Almost the same set of metabolites was responsible for the differences in urinary metabolic profiles of obese and lean *fa*/*fa* rats over a 40-week period, particularly microbial co-metabolites, metabolites of nicotinamide metabolism, and TCA metabolites. Dynamic changes in the levels of some metabolites between 12 and 21 weeks of age may be related to the development of the gut microbiome during adolescence in rats. The changes in the serum lipid profile of 40-week-old rats are consistent with previously published results in other experimental models of obesity. The significant decrease in tauro- and glycoconjugates of serum bile acids, as first analyzed in *fa*/*fa* rats, may also be related to gut microbiome changes induced by genetic obesity.

The main contribution of our study is to cover the information gap on the metabolomic status of elderly *fa*/*fa* rats. Our results demonstrated that this rat genetic model of obesity with leptin and insulin resistance is stable even at 10 months of age and is therefore suitable for long-term studies. Thus, Zucker fatty rats can be successfully applied to study disorders associated with older age, e.g., neurodegenerative diseases [20]. Moreover, since significant changes in urinary host-microbial co-metabolites and serum BAs were observed, it is desirable in the future to focus on microbiome characterization and its correlation with metabolomic data.

## Figures and Tables

**Table 1:** Body weight and selected metabolic parameters in fasted blood plasma of *fa*/*fa* and control rats at 12, 21, 32, and 40 weeks of age.

	12 w		21 w	
	Control	*fa*/*fa*	Control	*fa*/*fa*
Body weight (g)	292 ± 8	366 ± 8 ***	390 ± 6	514 ± 14 ****
Insulin (ng/mL)	0.27 ± 0.05	1.80 ± 0.45 *	0.46 ± 0.08	2.23 ± 0.28 ***
Leptin (ng/mL)	0.96 ± 0.11	45.12 ± 4.83 ****	2.42 ± 0.30	52.28 ± 9.93 **
Cholesterol (mmol/L)	1.37 ± 0.04	2.18 ± 0.07 ****	2.13 ± 0.04	8.20 ± 1.00 ***
Triglycerides (mmol/L)	0.86 ± 0.15	7.98 ± 1.26 ***	1.15 ± 0.16	7.85 ± 1.13 ***
	**31 w ^a^**		**40 w ^a^**	
	**Control**	***fa*/*fa***	**Control**	***fa*/*fa***
Body weight (g)	441 ± 9	572 ± 11 ****	456 ± 9	592 ± 18 ****
Insulin (ng/mL)	0.49 ± 0.06	1.24 ± 0.02 ****	0.27 ± 0.06	1.04 ± 0.11 ***
Leptin (ng/mL)	4.02 ± 0.61	46.55 ± 1.61 ****	3.74 ± 0.51	46.80 ± 1.90 ****
Cholesterol (mmol/L)	3.37 ± 0.27	7.83 ± 0.51 ****	2.08 ± 0.15	3.77 ± 0.22 ****
Triglycerides (mmol/L)	1.11 ± 0.08	4.30 ± 0.28 ****	1.24 ± 0.15	6.78 ± 1.15 **

Data are presented as means ± SEM. Statistical analysis was performed by Student’s *t*-test. * *p* < 0.05, ** *p* < 0.01, *** *p* < 0.001, **** *p* < 0.0001. ^a^—data acquired at 31 and 40 weeks have already been published in [20].

**Table 2:** Significantly changed metabolites in the urine of *fa*/*fa* and control rats at 12, 21, 32, and 40 weeks of age, detected by NMR.

Metabolite	12 w ∆ [%]	21 w ∆ [%]	32 w ∆ [%]	40 w ∆ [%]
1-Methylnicotinamide	41.96 **	56.08 ***	47.30 **	26.47 *
Trigonelline	34.25 ****	37.59 ***	22.51 *	29.94 *
4-PY	9.30	−25.82 ***	−31.56 ***	−30.72 ***
Hippurate	26.40 **	0.71	−47.86 ****	−56.30 ****
3-Indoxylsulfate	−35.72 **	−87.46 ****	−97.33 ****	−97.48 ***
*p*-Cresylglucuronide	20.38 ^x^	−12.62 *	−21.27 *	−26.16 ***
Putrescine	−42.39 ***	−43.73 ***	−40.00 *	−52.51 ****
Phenylacetylglycine	−53.02 ***	−43.67 **	−51.24 ***	−47.64 **
2-Oxoglutarate	48.84 **	59.37 ****	79.96 ****	66.64 ****
Fumarate	27.35	154.72 **	258.00 ***	186.05 ****
Citrate	34.38 **	72.34 ****	97.68 ****	95.19 ****
Malate	29.31 **	96.21 ***	122.81 ****	94.75 ****
Creatinine	−37.25 ****	−42.05 ****	−41.42 ****	−41.74 ****
Lactate	33.19 *	21.23 ^x^	30.57 ^x^	23.32 *
Taurine	13.64	−38.73 ^x^	−56.61 *	−53.14 ^x^
Formate	40.32	78.53	−58.75 *	−74.19 *
Choline	20.40 ^x^	33.06 **	57.38 **	47.17 *
Glycine	−11.95	21.76 ^x^	52.26 **	31.43 **
Alanine	−15.36 *	−3.63	23.06 ^x^	29.16 *
Orotate	1.34	−8.60	−42.82 **	−41.92 ***
Allantoin	−5.78	−8.25 **	−2.41	−5.14
Benzoate	−68.01 **	−68.62 **	−79.76 **	−74.31 ****
Dimethylsulfone	11.96	1.73	−18.18 *	−18.96 ^x^
Pseudouridine	−18.08 ****	−14.64 ***	−6.20 *	−12.92 ***
Methanol	42.37 **	45.58 ***	25.82 ^x^	24.47 ^x^
Methylsuccinate	−25.86 **	−13.50 *	−29.33 **	−42.39 ***
Doublet (1.25 ppm)	−29.25 ****	−30.21 ****	−29.31 ****	−32.52 ****
Lipids + keto-acids	−22.04 ***	−14.47 *	−24.10 **	−30.11 ****
Lipids	−73.66 ****	−71.75 ****	−65.34 ****	−64.21 ****

The results are expressed as the percentage change of normalized concentrations in *fa*/*fa* vs. controls. The statistical significance was analyzed by Student’s *t*-test. * *p* < 0.05, ** *p* < 0.01, *** *p* < 0.001, **** *p* < 0.0001, ^x^—trend with *p* < 0.1. 4-PY—1-Methyl-4-pyridone-3-carboxamide.

**Table 3:** Significantly changed metabolites in the urine of *fa*/*fa* and control rats during aging.

	21 w/12 w ∆ [%]		32 w/21 w ∆ [%]		40 w/32 w ∆ [%]	
Metabolite	*fa*/*fa*	Controls	*fa*/*fa*	Controls	*fa*/*fa*	Controls
1-Methylnicotinamide	−9.82	−23.37 *	−22.82 **	−16.69 *	−13.90	2.46
Trigonelline	−18.13 **	−19.68 **	−23.99 ***	−14.50 *	−1.72	−7.23
4-PY	−45.59 ***	−20.94 **	−23.98 **	−19.21 ****	−4.18	−2.42
Hippurate	−23.97 *	−8.04	−56.46 ****	−17.28 *	−18.47 *	−0.10
3-Indoxylsulfate	−89.25 ***	−37.52 **	−84.69	−33.49 **	−27.46	−22.61 *
*p*-Cresylglucuronide	−33.23 *	−11.96 *	−22.26 *	−15.19 *	−5.27	1.66
Phenylacetylglycine	−32.89 *	−36.96 *	−4.61	0.98	15.53	4.47
Putrescine	−18.22	−19.97 *	−10.52	−13.89 *	−12.58	9.69 *
2-Oxoglutarate	31.80 *	23.37	11.10	−1.09	−3.18	4.06
Fumarate	97.82 *	−4.55	21.30 *	−8.83	−0.49	22.85
Citrate	31.59 *	1.02	16.79 **	1.24	7.98	6.54
Malate	32.37 **	−14.05	5.32 *	−5.03	−1.84	10.91
Creatinine	−1.69	7.61 **	0.49	−0.19	8.30 **	8.17 **
Lactate	2.45	4.87	−5.98	−7.70	−13.79 *	−6.54
Taurine	18.24	90.47 *	−33.52	−6.31	−3.93	−10.94
Formate	147.56	64.11	−85.67 *	−40.20	−59.84	−38.95
Choline	−2.20	−10.84 **	15.28	3.11	−7.26	−0.83
Glycine	13.10	−16.52 **	17.24	−4.18	−6.33	9.99
Orotate	−30.49 **	−14.56	−25.25 *	2.00	−7.47	−4.88
Allantoin	−22.28 **	−19.38 ****	−0.65	−7.65 **	9.15 **	10.49 **
Dimethylsulfone	−18.39 **	−10.06 *	−19.04 *	3.63	−7.58	−2.63
Pseudouridine	−22.01 ***	−22.73 ****	4.13	−6.21 *	9.30 **	16.33 **
Methylsuccinate	7.74	−10.80	−25.91 **	−8.89	−6.90	16.99

The results are expressed as the percentage change of normalized concentrations in two consecutive time points for *fa*/*fa* and controls. The statistical significance was analyzed by paired *t*-test. * *p* < 0.05, ** *p* < 0.01, *** *p* < 0.001, **** *p* < 0.0001. 4-PY—1-Methyl-4-pyridone-3-carboxamide.

**Table 4:** Significantly changed metabolites in the serum of *fa*/*fa* and control rats at 40 weeks.

NMR Analysis		MS Analysis	
Metabolite	∆ [%]	Metabolite	∆ [%]
Citrate	95.42 ***	Valine	−38.96 ***
Fumarate	62.64 **	Leucine	−24.15 ***
Pyruvate	51.53 *	Glutamine	−11.82 **
Glucose	−16.37 *	Lysine	−25.52 ***
Arabinose	−23.20 **	Histidine	−20.00 ****
Lactate	40.60 *	Methylhistidine	−25.98 **
Glycerol	108.15 ***	Ornithine	−43.66 ****
Alanine	40.79 **	Carnitine	−28.52 ****
Asparagine	−34.16 ***	Acylcarnitine (C18:1)	25.77 *
Leucine	−14.94 **	Creatine	−19.97 ***
Valine	−22.18 ***	Deoxycytidine	−15.26 *
Lysine	−23.67 **	LysoPC (14:0)	86.18 ****
Histidine	−8.65 *	LysoPC (16:1)	96.84 ****
Creatine	−30.97 **	LysoPC (17:0)	−47.47 ****
Choline	−32.24 **	LysoPC (18:2)	−36.41 ***
Allantoin	67.43 ****	LysoPC (20:4)	43.79 **
3-Hydroxybutyrate	−32.40 **	PC (32:1)	206.98 ****
3-Hydroxyisobutyrate	−36.46 **	PC (35:4)	129.90 ***
Cytidine	−12.14 *	PC (36:2)	−44.20 ****
Thymidine	−14.25 *	PC (36:4)	20.61 *
Dimethylsulfone	26.97 *	PC (38:4)	39.17 ****
Ethanol	−17.75 *	PC (38:6)	37.92 **
Isoleucine + lipids	11.36 **	PC (40:6)	47.62 ****
Lipids + ketoacids	130.19 *	LysoPE (16:0)	36.87 *

The results are expressed as the percentage change of normalized concentrations in *fa*/*fa* vs. controls. The statistical significance was analyzed by Student’s *t*-test. * *p* < 0.05, ** *p* < 0.01, *** *p* < 0.001, **** *p* < 0.0001. PC-phosphatidylcholine, LysoPC—lysophosphatidylcholine, LysoPE—lysophosphatidylethanolamine.

**Table 5:** LC-MS targeted analysis of bile acids and neurotransmitters in serum.

Metabolite	∆ [%]	Metabolite	∆ [%]
*Bile acids*			
Cholic acid	89.58	Glycocholic acid	−71.20 ***
Ursodeoxycholic acid	−4.16	Tauroursodeoxycholic acid	−62.33 *
Hyodeoxycholic acid	−64.01 ^x^	Taurohyodeoxycholic acid	−67.55 **
Chenodeoxycholic acid	−1.97	Taurochenodeoxycholic acid	−46.64 ^x^
Deoxycholic acid	−45.32	Taurodeoxycholic acid	−50.49 ^x^
Glycoursodeoxycholic acid	−88.80 *	Taurocholic acid	−28.85
Glycohyodeoxycholic acid	−85.72 **	Taurolithocholic acid sulfate	−57.57 ^x^
Glycochenodeoxycholic acid	−74.45 **	β-muricholic acid	6.22
Glycodeoxycholic acid	−84.87 *		
*Neurotransmitters*			
Serotonin	48.36 ***	Kynurenine	−2.77
Tyramine	−13.03	Hydroxytryptophan	7.58
γ-Aminobutyric acid	1.32		

The results are expressed as the percentage change of normalized concentrations in *fa*/*fa* vs. controls. The statistical significance was analyzed by Student’s *t*-test. * *p* < 0.05, ** *p* < 0.01, *** *p* < 0.001, ^x^—trend with *p* < 0.1.

## Data Availability

The data presented in this study are available on request from the corresponding author. Ongoing studies with this particular data set are still in progress.

## Supplementary Materials

The following supporting information can be downloaded at: https://www.mdpi.com/article/10.3390/metabo13040552/s1, Table S1: The list of metabolites quantified in urine and serum by NMR; Figure S1: Representative ^1^H NMR spectrum of urine with quantified metabolites; Figure S2: Representative ^1^H NMR spectrum of serum with quantified metabolites; Table S2: The identification of significantly changed metabolites in untargeted LC-MS serum analysis; Figure S3: Score plots of the urine PLS-DA model.
