# Supplementary material for: Metabolomic Study of Aging in fa/fa Rats: Multiplatform Urine and Serum Analysis
Source: Metabolites. 2023 Apr 12;13(4):552. doi: 10.3390/metabo13040552 (PMC10142631; doi:10.3390/metabo13040552)
Supplement: Supplementary file 1 [file metabolites-13-00552-s001.zip › metabolites-2285756-supplementary.docx]

***SUPPLEMENTARY DATA***

Metabolomic study of aging in *fa/fa* rats: Multiplatform urine and serum analysis

Helena Pelantová ^1^, Petra Tomášová ^1,2^, Blanka Šedivá ^3^, Barbora Neprašová ^4^, Lucia Mráziková ^4^, Jaroslav Kuneš ^4,5^, Blanka Železná ^4^, Lenka Maletínská ^4^ and Marek Kuzma ^1^*

^1^ Institute of Microbiology, Czech Academy of Sciences, 142 20 Prague, Czech Republic

^2^ First Faculty of Medicine, Charles University and General University Hospital in Prague, 128 08 Prague, Czech Republic

^3^ Faculty of Applied Sciences, University of West Bohemia, 306 14, Pilsen, Czech Republic

^4^ Institute of Organic Chemistry and Biochemistry, Czech Academy of Sciences, 160 00 Prague, Czech Republic

^5^ Institute of Physiology, Czech Academy of Sciences, 142 20 Prague, Czech Republic

***** Correspondence: kuzma@biomed.cas.cz

**Table S1**: The list of metabolites quantified in urine and serum (with corresponding ^1^H and ^13^C chemical shifts) by NMR

|  | **metabolite** | **^1^H chemical shift [ppm]** | **^13^C chemical shift [ppm]** | **source** |
| --- | --- | --- | --- | --- |
| 1. | Lipoproteins CH_3_- | **0.80 – 0.87** (m) | 16.8 | S |
| 2. | Isoleucine | 0.94 (t), **1.01** (d) 1.47 (m), 1.98 (m) | 13.9, 17.4, 27.2 | S |
| 3. | Leucine | **0.96** (d), 0.97 (d), 1.71 (m), 1.72 (m), 3.74 (m) | 23.7, 24.8, 27.0, 42.6, 56.2 | S |
| 4. | Valine | 0.99 (d), **1.05** (d), 2.27 (m), 3.62 (d) | 19.5, 20.8, 31.9, 63.2 | U, S |
| 5. | 3-Hydroxyisobutyrate | **1.07** (d), 2.49 (m) | n.d. | S |
| 6. | Methylsuccinate | **1.09** (d), 2.15 (m), 2.53 (m), 2.64 (m) | 19.9 | U |
| 7. | 2-Oxoisovalerate | **1.13** (d) | 19.2 | S |
| 8. | Ethanol | **1.19** (t) | n.d. | S |
| 9. | 3-Hydroxybutyrate | **1.20** (d), 2.31 (dd), 2.40 (dd), 4.15 (m) | 24.6, 49.3, 68.4 | S |
| 10. | Lactate | **1.33** (d), **4.11** (q) | 22.9, 71.3 | U, S |
| 11. | Threonine | 1.33 (d), 3.59 (d), **4.26** (m) | 22.3, 63.3, 68.8 | S |
| 12. | Lysine | 1.46 (m), 1.51 (m), 1.73 (m), 1.91 (m), **3.03** (t), 3.76 (m) | 24.2, 29.5, 32.7, 41.8, 57.3 | S |
| 13. | Alanine | **1.49** (d), 3.79 (q) | 19.0, 53.3 | U, S |
| 14. | Putrescine | **1.78** (m), 3.06 (m) | 26.7, 41.7 | U |
| 15. | Acetate | **1.93** (s) | 26.1 | U, S |
| 16. | Glutamine | 2.14 (m), **2.46** (m) | 29.1, 33.7 | S |
| 17. | Methionine | 2.14 (s), **2.65** (t) | 31.7 | S |
| 18. | Acetone | **2.23** (s) | n.d. | S |
| 19. | *p*-Cresyl glucuronide | **2.30** (s), 5.08 (d), 7.06 (m), 7.23 (m) | 22.4, 103.4, 119.6, 133.1 | U |
| 20. | Pyruvate | **2.38** (s) | 29.3 | S |
| 21. | Malate | 2.39 (dd), **4.31** (dd) | 45.3, 73.1 | U |
| 22. | Succinate | **2.42** (s) | 36.8 | U |
| 23. | 2-Oxoglutarate | **2.45** (t), 3.01 (t) | 33.4, 38.6 | U |
| 24. | Citrate | 2.54 (d), **2.66** (d) | 48.6 | U, S |
| 25. | Dimethylamine | **2.72** (s) | 37.4 | U |
| 26. | Asparagine | **2.87** (dd), 2.95 (dd) | n.d. | S |
| 27. | Trimethylamine | **2.89** (s) | 47.5 | U |
| 28. | Dimethylglycine | **2.94** (s) | 46.3 | U |
| 29. | Creatine | **3.04** (s), 3.936 (s) | 39.7, 56.7 | S |
| 30. | Creatinine | **3.05** (s), 4.07 (s) | 33.0, 59.2 | U |
| 31. | *Cis*-aconitate | **3.14** (m), 5.77 (m) | 46.4, 127.6 | U |
| 32. | Dimethylsulfone | **3.16** (s) | 44.4 | S |
| 33. | Choline | **3.20** (s), 4.05 (m) | 56.6, 58.8 | U, S |
| 34. | Carnitine | **3.23** (s) | 56.8 | U, S |
| 35. | Taurine | **3.28** (t), **3.44** (t) | 38.2, 50.4 | U, S |
| 36. | Methanol | **3.37** (s) | 51.8 | U |
| 37. | Glycerol | 3.56 (dd), **3.66** (dd) | 65.3 | S |
| 38. | Glycine | **3.57** (s) | 44.4 | U |
| 39. | Phenylacetylglycine | 3.68 (m), 3.76 (m), 7.36 (m), 7.37 (m), **7.42** (m) | 45.0, 46.3, 130.1, 131.7, 132.1 | U |
| 40. | Hippurate | 3.98 (d), 7.55 (m), **7.64** (m), 7.84 (m) | 46.7, 129.9, 131.6, 135.0 | U |
| 41. | Trigonelline | 4.44 (s), 8.09 (m),8.84 (m), 8.85 (m), **9.13** (s) | 51.0, 130.5, 147.5, 148.6, 148.7 | U |
| 42. | 1-Methylnicotinamide | 4.48 (s), 8.19 (m), 8.91 (m), 8.97 (m), **9.28** (s) | 51.4, 130.9, 146.4, 147.9, 150.1 | U |
| 43. | Arabinose | **4.52** (d), 5.25 (d) | 95.1, 99.7 | U, S |
| 44. | Ascorbate | **4.53** (d) | 81.4 | U |
| 45. | Glucose | 3.25 (dd), 3.41 (m), 3.47 (m), 3.50 (dd), 3.54 (dd), 3.72 (m), 3.77 (dd), 3.84 (m), 3.90 (dd), 4.66 (d), **5.25** (d) | 63.4, 63.6, 72.5, 74.3, 75.6, 77.0, 78.6, 78.8, 94.8, 98.6 | U, S |
| 46. | Mannose | 4.91 (d), **5.19** (d) | 96.3, 96.9 | S |
| 47. | Allantoin | **5.39** (s) | 66.1 | U, S |
| 48. | Cytidine | **6.07** (d), 7.83 (d) | n.d. | U |
| 49. | Orotate | **6.20** (s) | 104.2 | U |
| 50. | Thymidine | **6.28** (t) | n.d. | U |
| 51. | Fumarate | **6.53** (s) | 138.1 | U, S |
| 52. | 1-Methyl-4-pyridone-3-carboxamide | 6.70 (d), 7.97 (dd), **8.55** (d) | 122.9, 142.2, 149.6 | U |
| 53. | Tyrosine | **6.91** (m), 7.20 (m) | 118.7, 133.6 | S |
| 54. | Histidine | **7.10** (s), 7.90 (s) | n.d. | S |
| 55. | Tryptophan | 7.20 (m), 7.29 (m), 7.55 (m), **7.74** (m) | 121.3 | S |
| 56. | Phenylalanine | 7.33 (m), **7.43** (m) | 131.9, 132.3 | S |
| 57. | 3-Indoxylsulfate | 7.51 (m), **7.71** (m) | 114.7 | U |
| 58. | Pseudouridine^x^ | **7.68** (s) | 144.5 | U |
| 59. | Formate | **8.46** | 172.5 | U, S |

The table lists all metabolites quantified in urine (U) and serum (S); the signals used for metabolite quantification are in bold. Signal multiplicity is marked as follows: (s)-singlet, (d)-doublet, (t)-triplet, (dd)‑doublet of doublets, (q)-quartet, (m)-multiplet; n.d. – signals not detected; ^x^-tentative assignment.


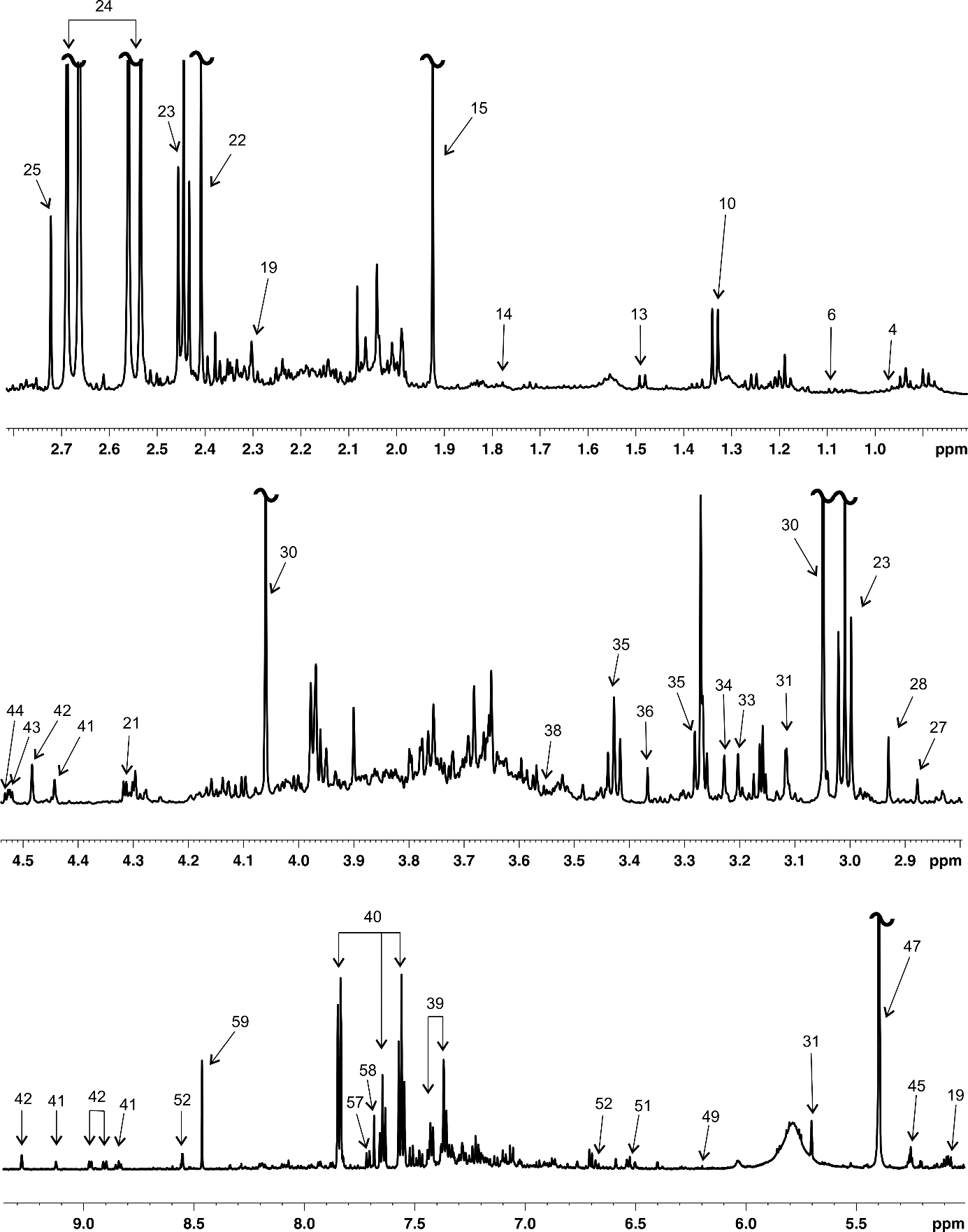


**Figure S1.** Representative ^1^H NMR spectrum of urine with quantified metabolites. Metabolite assignments for the numbers are given in Table S1.


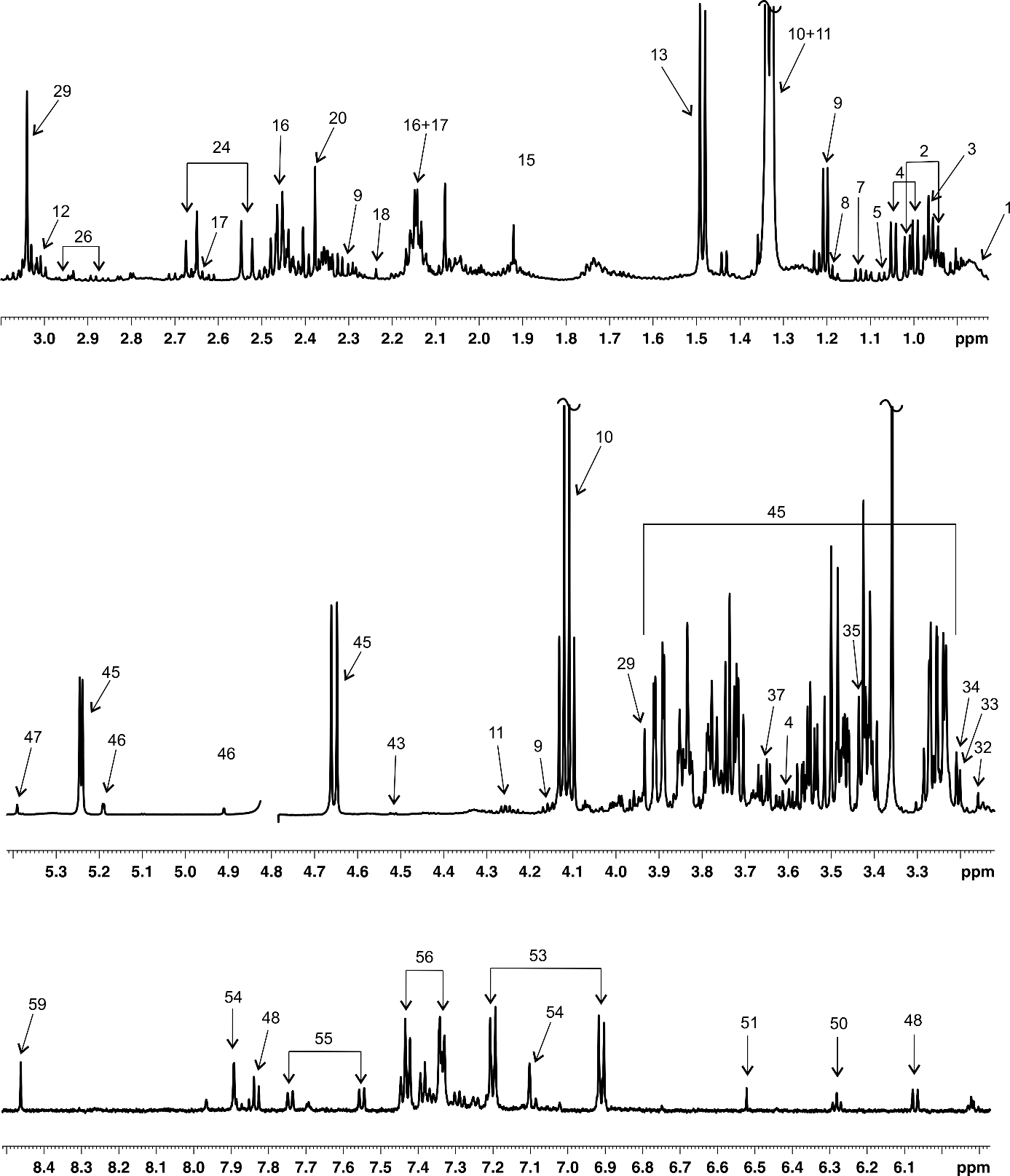


**Figure S2.** Representative ^1^H NMR spectrum of serum with quantified metabolites. Metabolite assignments for the numbers are given in Table S1.

**Table S2**: The identification of significantly changed metabolites in untargeted LC-MS serum analysis

| **Metabolite** | **RT [min]** | **m/z** |
| --- | --- | --- |
| Valine | 6.0 | 118.0863 |
| Leucine | 6.8 | 132.1019 |
| Glutamine | 14.7 | 147.0764 |
| Lysine | 23.2 | 147.1128 |
| Histidine | 22.0 | 156.0768 |
| Methylhistidine | 22.2 | 170.0924 |
| Ornithine | 23.4 | 133.0972 |
| Carnitine | 10.4 | 162.1125 |
| Acylcarnitine (C18:1) | 3.5 | 426.3578 |
| Creatine | 13.5 | 132.0768 |
| Deoxycytidine | 5.3 | 228.0979 |
| LysoPC(14:0) | 4.0 | 468.3085 |
| LysoPC(16:1) | 3.9 | 494.3241 |
| LysoPC(17:0) | 3.7 | 510.3554 |
| LysoPC(18:2) | 3.7 | 520.3398 |
| LysoPC(20:4) | 3.5 | 544.3398 |
| PC(32:1) | 2.4 | 732.5538 |
| PC(35:4) | 2.5 | 768.5538 |
| PC(36:2) | 2.4 | 786.6007 |
| PC(36:4) | 2.2 | 782.5694 |
| PC(38:4) | 2.3 | 810.6007 |
| PC(38:6) | 2.2 | 806.5694 |
| PC(40:6) | 2.3 | 834.6007 |
| LysoPE(16:0) | 4.8 | 454.2928 |

PC ‑ phosphatidylcholine, LysoPC - lysophosphatidylcholine, LysoPE - lysophosphatidylethanolamine.


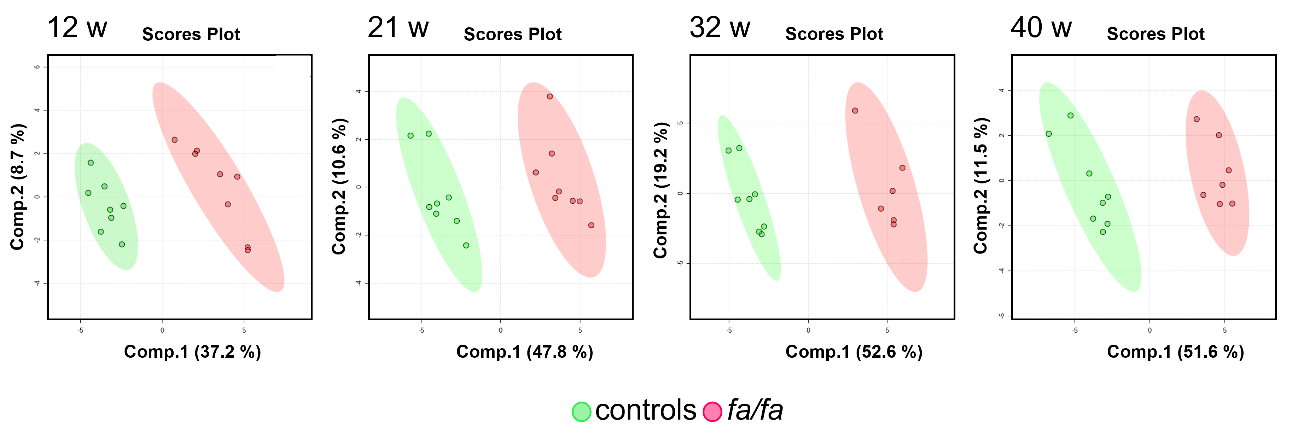


**Figure S3**. Scores plots of the PLS-DA model of urine samples from *fa/fa* and control rats at 12, 21, 32, and 40 weeks. The LOOV results for 2 principal components: accuracy=1, R2=0.97, Q2=0.87 at 12 weeks; accuracy=1, R2=0.97, Q2=0.88 at 21 weeks; accuracy=1, R2=0.98, Q2=0.95 at 32 weeks; accuracy=1, R2=0.97, Q2=0.92 at 40 weeks. *Fa/fa* group is marked in red, control group in green.
